# The frequency and quality of delirium documentation in discharge summaries

**DOI:** 10.1186/s12877-021-02245-3

**Published:** 2021-05-12

**Authors:** Victoria L Chuen, Adrian C.H Chan, Jin Ma, Shabbir M.H Alibhai, Vicky Chau

**Affiliations:** 1 Faculty of Medicine, University of Toronto, Ontario Toronto, Canada; 2 Faculty of Medicine, McMaster University, Ontario Hamilton, Canada; 3 Faculty of Medicine, University of Saskatchewan, Saskatoon, Saskatchewan Canada; 4 Biostatistics Research Unit, University Health Network, Toronto, Ontario Canada; 5 Division of General Internal Medicine and Geriatrics, Department of Medicine, University Health Network, Toronto, Ontario Canada; 6 Division of General Internal Medicine and Geriatrics, Department of Medicine, Sinai Health System, Ontario Toronto, Canada

**Keywords:** Delirium, Discharge summary, Quality, Documentation, Geriatrics

## Abstract

**Background:**

The National Institute for Health and Care Excellence recommends documenting all delirium episodes in the discharge summary using the term “delirium”. Previous studies demonstrate poor delirium documentation rates in discharge summaries and no studies have assessed delirium documentation quality. The aim of this study was to determine the frequency and quality of delirium documentation in discharge summaries and explore differences between medical and surgical services.

**Methods:**

This was a multi-center retrospective chart review. We included 110 patients aged ≥ 65 years identified to have delirium during their hospitalization using the Chart-based Delirium Identification Instrument (CHART-DEL). We assessed the frequency of any delirium documentation in discharge summaries, and more specifically, for the term “delirium”. We evaluated the quality of delirium discharge documentation using the Joint Commission on Accreditation of Healthcare Organization’s framework for quality discharge summaries. Comparisons were made between medical and surgical services. Secondary outcomes included assessing factors influencing the frequency of “delirium” being documented in the discharge summary.

**Results:**

We identified 110 patients with sufficient chart documentation to identify delirium and 80.9 % of patients had delirium documented in their discharge summary (“delirium” or other acceptable term). The specific term “delirium” was reported in 63.6 % of all delirious patients and more often by surgical than medical specialties (76.5 % vs. 52.5 %, *p* = 0.02). Documentation quality was significantly lower by surgical specialties in reporting delirium as a diagnosis (23.5 % vs. 57.6 %, *p* < 0.001), documenting delirium workup (23.4 % vs. 57.6 %, *p* = 0.001), etiology (43.3 % vs. 70.4 %, *p* = 0.03), treatment (36.7 % vs. 66.7 %, *p* = 0.02), medication changes (44.4 % vs. 100 %, *p* = 0.002) and follow-up (36.4 % vs. 88.2 %, *p* = 0.01).

**Conclusions:**

The frequency of delirium documentation is higher than previously reported but remains subpar. Medical services document delirium with higher quality, but surgical specialties document the term “delirium” more frequently. The documentation of delirium in discharge summaries must improve to meet quality standards.

**Supplementary Information:**

The online version contains supplementary material available at 10.1186/s12877-021-02245-3.

## Introduction

Delirium is an acute and fluctuating disturbance in attention and cognition, precipitated by a physiologic, pharmacologic or environmental insult [1]. It affects 11–51 % and 29–64 % of surgical and medical patients, respectively [2]. Older adults are at risk for delirium, which is associated with increased length of stay [3–5], institutionalization [3, 6], mortality [6–8] and cognitive impairment [7, 9].

 The National Institute for Health and Care Excellence (NICE) established quality standards for the prevention, diagnosis and management of delirium. These standards recommend delirium be communicated to the patient’s primary care provider (PCP) using the term “delirium” in the discharge summary [10].

The frequency of delirium documentation in discharge summaries has varied in previous studies, with documentation of delirium (symptoms or diagnosis) ranging from 7 to 44 % for patients identified to have delirium either through clinical diagnosis or retrospective chart review. Only a small number of patients (3–16 %) had delirium documented specifically as a diagnosis in the discharge summary [11–16], and the term “delirium” was used in 40 % of cases [12]. Higher delirium documentation rates are associated with structured discharge summaries, female patients, and greater delirium severity [12].

We did not find studies describing the quality of delirium documentation in either medical or surgical discharge summaries; however, literature suggests the quality of discharge summaries across various specialties is subpar [17–20]. Poor discharge documentation is associated with hospital readmissions [18, 21] and adverse events [22]. Given 32–84 % of delirium cases persist after discharge [8, 23, 24], the discharge summary should be used to highlight necessary follow-up by the PCP or other consultants.

Our primary objectives were to characterize delirium documentation in discharge summaries by assessing its frequency and quality, and to compare documentation between medical and surgical services. Secondly, we aimed to identify factors influencing delirium documentation.

## Methods

We employed a retrospective chart-review design. Patients aged ≥ 65 years admitted to any one of three academic tertiary acute care hospitals by a medical (General Internal Medicine/Clinical Teaching Unit, Hospitalist, Neurology, Cardiology) or surgical (General Surgery, Orthopedic Surgery, Cardiac Surgery, Neurosurgery) service between 1 April and 30 June 2016 were screened for eligibility. Patients transferred from an intensive care unit (ICU) to one of the above services were included. Admissions restricted to the ICU, patients without a discharge summary and who died in hospital were excluded. Our study was approved by each institution’s research ethics board prior to commencement with a waiver for individual patient consent.

### Screening for delirium

We applied the validated Chart-Based Delirium Identification Instrument (CHART-DEL) (sensitivity 74 %, specificity 83 %) to identify delirium [25]. An assessment was made to determine the probability of delirium, (for further details on this methodology, see Additional file 1). To increase sensitivity, we included only “definite” or “probable” cases using CHART-DEL criteria. We also excluded any cases of delirium identified through the CHART-DEL, that were not recognized by a physician during the patient’s hospitalization (*n* = 2). Some cases of delirium were diagnosed by a consulting service, but not recognized by the attending team; we opted to include these cases of delirium given they had received a formal diagnosis for delirium. Parallel ratings were completed for at least ten charts between an expert rater (e.g. geriatrician) and each research assistant to ensure congruency in the CHART-DEL classification [26].

### Data collection

Baseline demographic data was collected on each patient. Additionally, we collected data on delirium episodes, including its time of onset, work-up, diagnosis, management, resolution and duration.

### Frequency of delirium documentation in the discharge summary

We assessed each discharge summary for delirium documentation. Delirium was considered documented if it was reported anywhere in the discharge summary. This included the use of “delirium” specifically, other terms such as “confusion”, and those listed as synonymous with delirium in Appendix II of the CHART-DEL manual [26].

### Quality of delirium documentation in the discharge summary

There are no validated tools to assess the quality of delirium documentation. Using the standards set by the Joint Commission on Accreditation of Healthcare Organizations (JCAHO) [19], designed to guide quality discharge documentation, we identified criteria to assess the quality of delirium documentation (Table 1**)** using definitions by Kind et al. [19] The tool was approved by a geriatric expert panel and was applied to each discharge summary. Comparisons were made between medical and surgical groups.

**Table 1:** Consensus criteria to assess the frequency and quality of delirium documentation in discharge summaries

JCAHO requirement^a^	Consensus criteria specific for delirium
**Reason for Hospitalization**^b^	
Chief complaint AND/OR HPI	Documentation of delirium as a chief complaint Documentation of the HPI for delirium episode
**Significant Findings**	
Primary diagnosis	Documentation of delirium as a primary diagnosis (if delirium was the reason for admission) Documentation of delirium as a secondary diagnosis^c^
**Procedures Performed**	
Hospital course AND/OR Hospital consults AND/OR Hospital procedures	Documentation of delirium in a problem list format Documentation of the onset of delirium Documentation of the cause of delirium Documentation of any specialist’s consultations in managing the delirium Documentation of completed delirium work-up investigations Documentation of received treatments for the primary cause of delirium
**Patient’s Condition at Discharge**	Documentation of current state of delirium (resolved or not) Documentation of patient’s functional status at discharge
**Patient/Family Instructions**	
Discharge medications AND/OR Activity orders AND/OR Therapy orders AND/OR Dietary instructions AND/OR Plans for medical follow-up	Documentation of counselling/education provided to patient’s family or caregiver regarding delirium Documentation of medication changes, as relevant to delirium Documentation of rationale for medication changes for delirium Documentation of recommended medication follow-up for delirium Documentation of recommended cognitive follow-up for delirium Documentation of any referrals/follow-up with specialists for delirium Documentation of patient’s primary care provider
**Physician Signature**	Documentation of electronic signature of discharge summary author

^a^As defined by Kind et al. [19]

^b^If symptoms of delirium were part of the patient’s chief complaint or HPI, it was expected to be documented in the discharge summary as such

^c^Documenting delirium as a secondary diagnosis was expected for cases where delirium was not deemed to be the primary reason for admission

### Data analysis

We performed statistical analyses using R, version 3.6.0. Descriptive statistics were used to analyze patient characteristics, delirium characteristics, and discharge summary documentation. Analysis of delirium quality measures deserves special mention. If certain quality components were not completed during hospitalization (e.g. delirium work-up), the patient would be omitted from the proportion calculation for that given component. This was because we would not expect the attending team to document a component that they did not complete. Similarly, if no consultant or medication changes were applicable with respects to delirium, we would not expect documentation. Further detail on the definitions for each quality component is available in Additional File 2. However, all patients were included in the proportion calculation to assess whether delirium was documented anywhere in the discharge summary, as a diagnosis, or listed in a problem list.

The dataset was analyzed collectively and grouped to explore the *a priori* hypothesis that delirium documentation would differ between medical and surgical services. A comparison between groups was done using the Student’s t-test for continuous variables and Chi-squared or Fisher’s exact test for categorical variables. The level of significance was set at a p-value of < 0.05.

Univariate logistic regression modelling was used to determine the relationship between documentation of “delirium” specifically in the discharge summary, and various factors based on *a priori* hypotheses. Some variables were selected based upon findings from previous studies [12, 27] and others based on consensus between researchers.

### Sample size

Based on our *a priori* hypotheses that the frequency and quality of delirium documentation would differ between medical and surgical services, we aimed for a sample size that would provide adequate power for this. Assuming alpha = 0.05 and power = 0.90 with a difference in delirium documentation of 30 %, we aimed for a sample of 56 patients in each group. We achieved 59 medical patients, and 51 surgical patients.

## Results

Of 1 168 patient charts screened, 118 patients had “definite” or “probable” delirium as identified through the CHART-DEL. We included 110 patients. A total of 8 charts were omitted, 6 charts due to incomplete data abstraction and 2 charts because delirium was not recognized by any physician in the care team. Patients had a mean age of 79.6 years and 55.5 % were male (Table 2). We captured patients with all types of delirium, with 30.9 % hypoactive, 25.5 % hyperactive and 43.6 % mixed cases.

**Table 2:** Study population demographic information

Characteristic	Total (*n* = 110)	Medicine (*n* = 59)	Surgery (*n* = 51)	*p*-value^a^
**Age**, mean (SD)	79.6 (8.4)	81.0 (7.7)	78.0 (9.0)	0.06
**Male sex**, n (%)	61 (55.5)	32 (54.2)	29 (56.9)	0.93
**English as primary language**^b^, n(%)	72 (66.7)	39 (68.4)	33 (64.7)	0.84
**Baseline cognition**^c^, n (%)				0.07
Dementia	28 (25.5)	20 (33.9)	8 (15.7)	
Mild cognitive impairment	4 (3.6)	2 (3.4)	2 (3.9)	
Psychiatric illness	14 (12.7)	5 (8.5)	9 (17.6)	
Other cognitive impairment	18 (16.4)	12 (20.3)	6 (11.8)	
No impairment	46 (41.8)	20 (33.9)	26 (51.0)	
**Baseline functional status**^b d^, n (%)				< 0.001
Independent	28 (28.9)	10 (19.2)	18 (40.0)	
Impairment in 1–2 domains	44 (45.4)	19 (36.5)	25 (55.6)	
Dependent in ≥ 3 domains	25 (25.8)	23 (44.2)	2 (4.4)	
**Pre-admission residence**^b e^, n (%)				0.03
Home	84 (77.1)	40 (67.8)	44 (88.0)	
Nursing home/LTC	13 (11.9)	11 (18.6)	2 (4.0)	
Retirement home	9 (8.3)	5 (8.5)	4 (8.0)	
Other	3 (2.7)	3 (5.1)		
**Pre-admission social supports**^b f^, n (%)				0.07
Alone	22 (26.5)	8 (19.5)	14 (33.3)	
Alone with external supports	12 (14.5)	9 (22.0)	3 (7.1)	
Living with family	32 (38.6)	13 (31.7)	19 (45.2)	
Living with family with external supports	17 (20.5)	11 (26.8)	6 (14.3)	
**History of previous delirium**^b^, n (%)	32 (33.3)	18 (36.7)	14 (29.8)	0.61
**Charlson Comorbidity Index**, median [IQR]	2.0 [1.0–4.0]	2.0 [1.0–4.0]	2.0 [1.0–3.0]	0.18
**Number of home medications**, mean (SD)	8.9 (4.9)	9.3 (5.5)	8.4 (4.1)	0.31
**Length of stay**, median days [IQR]	8.5 [5.0, 22.8]	7.0 [3.0-18.5]	13.0 [7.0-28.5]	0.01

*LTC* long term care

^a^Comparing medicine vs. surgery

^b^Indicates missing data (n). Language (n = 2), Functional status (n = 13), Residence (n = 1), Supports (n = 1), Previous delirium (n = 14). Proportions were calculated without missing data

^c^Baseline cognition was assessed based on the patient’s documented past medical history. Psychiatry illness (inclusive of any DSM5 diagnosis), other cognitive impairment (included documentation of memory issues/decline without formal diagnosis of mild cognitive impairment or dementia)

^d^Baseline functional status was assessed based on their independence with basic and instrumental activities of daily living (BADLs, IADLs). Independent (no BADL or IADL impairment), impairment (assistance with one to two BADLs or one to two IADLs), dependent (assistance with more than two BADLs)

^e^Other category included complex continuing care, long-term care unit in hospital

^f^Baseline social supports were evaluated for patients who resided at home (n = 84) and excluded those living in institutionalized care. External supports included both government and privately funded services (e.g. personal support worker, home care)

### Frequency of delirium documentation in the discharge summary

**Fig. 1:**
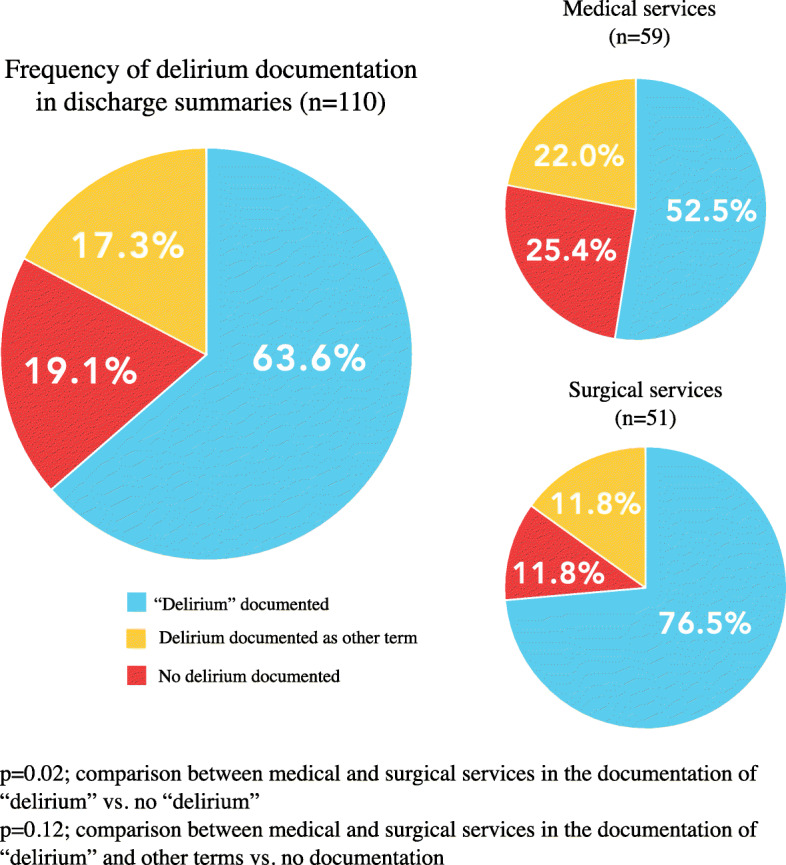
Frequency of delirium documentation in discharge summaries belonging to delirious patients identified using the CHART-DEL

The overall documentation rate of delirium in discharge summaries was 80.9 % (“delirium” and other terms accepted). Amongst discharge summaries with delirium documentation (*n* = 89), most used the word “delirium” (*n* = 70, 78.7%) compared to another acceptable term (*n* = 19, 21.3 %). The overall frequency of delirium documentation (“delirium” and other terms) did not differ significantly between medical and surgical services (74.6 % vs. 88.2 %, *p* = 0.12, Fig. 1).

### Quality of delirium documentation in the discharge summary

The specific term “delirium” was documented in 63.6 % of discharge summaries (*n* = 70) belonging to patients identified to have delirium through chart review (*n* = 110). When comparing between services, surgical services documented “delirium” more than medical services (76.5 % vs. 52.5 %, *p* = 0.02) (Fig. 1). In cases where delirium symptoms developed prior to admission (*n* = 47), 66.0 % of discharge summaries documented it within the chief complaint or HPI.

Across discharge summaries, only 30.9 % had “delirium” documented as a diagnosis. Not all individuals had delirium as their primary reason for admission, especially since delirium is often secondary to a comorbid process. When delirium was the stated reason for admission (*n* = 38), 23 patients (60.5 %) had delirium appropriately documented as a primary diagnosis in the discharge summary. For all other cases (*n* = 72), 23 patients (31.9 %) documented it as a secondary diagnosis. Acceptable documentation as a secondary diagnosis included having delirium mentioned in a problem list or dedicated paragraph discussing delirium.

When describing delirium within the hospital course, medical services more often documented the underlying etiology, work-up and treatment of delirium. A problem list identifying delirium was included in 31.8 % of charts, with significantly higher rates by medical services (*p* = 0.01). The onset of delirium and consultant involvement was documented in 63.6% and 38.2 % of all discharge summaries respectively (Table 3).

**Table 3:** Quality components of delirium documentation in discharge summaries

Quality component n (%)^a^	Total (*n* = 110)	Medicine (*n* = 59)	Surgery (*n* = 51)	*p*-value^b^
**Documented as chief complaint or HPI**	31/47 (66.0)	29/42 (69.0)	2/5 (40.0)	0.32
**Documented as diagnosis**				
“Delirium”	34/110 (30.9)	22/59 (37.3)	12/51 (23.5)	0.18^c^
“Delirium” or other term	46/110 (41.8)	34/59 (57.6)	12/51 (23.5)	< 0.001^d^
Not documented	64/110 (58.2)	25/59 (42.4)	39/51 (76.5)	
**Documented as 1º diagnosis**^e^	23/38 (60.5)	21/34 (61.8)	2/4 (50.0)	1.00
**Documented as 2º diagnosis**^e^	23/72 (31.9)	13/25 (52.0)	10/47 (21.3)	0.02
**Documented in problem list**^e^	35/110 (31.8)	26/59 (44.1)	9/51 (17.6)	0.01
**Documented delirium onset**	70/110 (63.6)	41/59 (69.5)	29/51 (56.9)	0.24
**Documented delirium etiology**	51/84 (60.7)	38/54 (70.4)	13/30 (43.3)	0.03
**Documented consulting service involvement**	21/55 (38.2)	9/27 (33.3)	12/28 (42.9)	0.65
**Documented delirium work-up**	45/106 (42.5)	34/59 (57.6)	11/47 (23.4)	0.001
**Documented delirium treatment**	47/84 (56.0)	36/54 (66.7)	11/30 (36.7)	0.02
**Documented delirium status at discharge**	56/84 (66.7)	30/46 (65.2)	26/38 (68.4)	0.94
**Documented functional status at discharge**	37/110 (33.6)	17/59 (28.8)	20/51 (39.2)	0.34
**Documented relevant medication changes**	20/25 (80.0)	16/16 (100.0)	4/9 (44.4)	0.002
**Documented reason for medication changes**	14/25 (56.0)	12/16 (75.0)	2/9 (22.2)	0.02
**Documented patient or family instructions**	52/110 (47.3)	23/59 (40.0)	29/51 (56.9)	0.06
**Documented any follow-up recommendations**	19/28 (67.9)	15/17 (88.2)	4/11 (36.4)	0.01
**Documented follow-up for psychoactive medications**	9/12 (75.0)	9/9 (100.0)	0/3 (0.0)	0.01
**Documented follow-up for cognitive status**	5/11 (45.5)	3/5 (60.0)	2/6 (33.3)	0.57
**Documented follow-up with specialist**	8/10 (80.0)	6/6 (100.0)	2/4 (50.0)	0.13
**Copied to primary care provider**	89/110 (80.9)	54/59 (91.5)	35/51 (68.6)	0.01
**Signed by author**	110/110 (100.0)	59/59 (100.0)	51/51 (100.0)	

Denominators for each quality component differed, depending on its applicability to the patient. For instance, if no changes were made to psychoactive medications, then this would not be applicable and expected to be documented in the discharge summary

^a^Components based on the JCAHO requirements for a quality discharge summary, as defined by Kind et al. [19] and made specific for delirium. See Additional File 2 for full definitions developed specifically for delirium

^b^Comparing medicine vs. surgery

^c^When compared to grouped data from other term and no delirium documentation

^d^When compared to no delirium documentation

^e^Documentation of the term “delirium” or other term

The resolution status of delirium and functional status at discharge was documented in 66.7 and 33.6 % of discharge summaries, respectively. Medical services documented medication changes in 100 % of applicable discharge summaries, whereas surgical services in 44.4 % (*p* = 0.002).

About half (47.3 %) of discharge summaries included patient or family instructions pertaining to delirium. When any delirium follow-up was recommended (*n* = 28) (e.g. cognitive testing, medication review or specialist appointment), medical services had significantly higher documentation rates than surgical services (88.2 % vs. 36.4 %, *p* = 0.01).

### Factors impacting delirium documentation in the discharge summary

The strongest univariate predictor for documenting “delirium” in the discharge summary, was being admitted under a surgical service (OR 2.94, 95 % CI [1.29–6.70]). Negative predictors included patients who presented to hospital with symptoms of delirium (OR 0.33, 95 % CI [0.15–0.73]) and those who had delirium as their presenting reason for admission to hospital (OR 0.41, 95 % CI [0.18–0.93]) (Table 4).

**Table 4:** Variables affecting the documentation of “delirium” specifically in the discharge summary

Variable	Univariate odds ratio (95 % CI)
**Admitted to surgical service**	2.94 (1.29–6.70)
**Male sex**^a^	1.21 (0.55–2.63)
**Formal diagnosis of delirium made**^b^	2.27 (0.14–37.46)
**No history of dementia**	0.96 (0.39–2.36)
**Presence of delirium symptoms prior to admission**	0.33 (0.15–0.73)
**Delirium as the reason for admission to hospital**	0.41 (0.18–0.93)
**Delirium Type**	
Hypoactive delirium	0.71 (0.29–1.78)
Hyperactive delirium	0.90 (0.34–2.40)
Mixed	Reference level
**CCI, per 1-point increase**	0.89 (0.74–1.08)
**Structured discharge summary**^a c^	0.55 (0.18–1.70)
**LOS, per day**	1.03 (0.999–1.06)
**Duration of delirium**^d^	0.996 (0.98–1.01)
**Discharge summary author**^e^	
Early trainee	0.73 (0.29–1.85)
Allied Health	4.62 (0.93–22.89)
Staff	0.65 (0.21–2.04)
Senior Resident	Reference level

*CCI* Charlson Comorbidity Index, *LOS* length of stay

^a^Variable identified previously to affect the rate of delirium documentation in discharge summaries [12, 27]. All other variables were selected based on consensus between researchers

^b^Diagnosis made by any involved physician using specifically the term “delirium”

^c^Defined as having standardized discharge summary headings (e.g. Chief complaint, HPI, Medications…etc)

^d^Defined as proportion of hospital stay

^e^Early trainee = medical students, Year One residents; Senior trainees = Year Two and higher residents, fellows; Allied health = physician assistants, nurse practitioners.

## Discussion

Our study allowed us to perform a quality assessment of discharge summary documentation for delirium in a small sample size (*n* = 110). Of hospitalized patients with clear evidence of delirium documented in their chart, 80.9 % had some level of delirium documentation in their discharge summaries. The term “delirium” was documented more frequently by surgical than medical services. However, medical services demonstrated a higher quality of delirium documentation, specifically with documenting delirium as a diagnosis (“delirium” or other terms), inclusion in a problem list, and the delirium work-up, etiology, treatment, related medication changes and follow-up plan.

### Frequency of delirium documentation in the discharge summary

Our results demonstrate a substantial improvement in the frequency of delirium documentation in discharge summaries from previous [11, 12, 28]. This higher rate of delirium discharge documentation could be explained by our methodology. While other studies identified delirium solely through clinical assessments [11, 12], our use of the CHART-DEL is inherently linked to improved delirium documentation throughout the admission, which could in turn increase documentation in the discharge summary.

### Quality of delirium documentation in the discharge summary

To our knowledge, our study is the first to assess the quality of delirium documentation in discharge summaries, and our results identify areas needing improvement. An important component of quality documentation is to specifically document “delirium”, as recommended by the NICE quality standards for delirium. Additionally, an expert consensus developed by a multi-disciplinary panel recommends the use of “delirium” when all criteria set by the fifth version of the Diagnostic and Statistical Manual are met. The term “acute encephalopathy” should be used to refer to the underlying processes in the brain, which result in delirium [29]. Although surgical services frequently documented “delirium”, remaining quality components were lacking. For instance, delirium was infrequently documented as a diagnosis. This may reflect that it is regarded as a postoperative complication, rather than a diagnosis with its own long-term sequelae and required follow-up [30].

Surgical services poorly documented medication changes or follow-up instructions relating to delirium (Table 3). This is not surprising as it has previously been shown that only 12.4 % of delirious patients started on an antipsychotic in hospital have discontinuation instructions documented in the discharge summary [31]. The effect of discharge prescription instructions on patient outcomes or PCP prescribing patterns has not been studied to our knowledge; however, PCPs do appreciate pharmacotherapeutic advice and documented reasons for medication changes upon discharge [32].

A qualitative study investigating barriers to quality orthopedic discharge summaries suggested improvements could be made through use of discharge summary templates and co-authorship, given the multiple clinicians involved with differing expertise [33–35]. However, often no consultant is involved in delirium management, thus other strategies are needed to improve documentation.

The documentation of delirium in discharge summaries remains subpar. Our study was completed at an academic centre where most discharge summaries are completed by trainees. Effective strategies to improve the quality of discharge summaries include formalized teaching curriculum or workshops [36–38], quality improvement projects [39, 40], and performing audits with feedback sessions [41]. These strategies could be implemented to improve delirium documentation during residency and thereafter.

### Factors impacting the frequency of delirium documentation in the discharge summary

Surgical services demonstrated a higher quality of delirium documentation with respect to using the specific term “delirium” (Fig. 1). In our univariable model, authorship by a surgical service was a positive predictor for having “delirium” documented, which perhaps demonstrates recognition of delirium as a relevant and common post-operative complication (Table 4).

Delirium symptoms at the time of presentation and delirium being the reason for admission to hospital were both variables associated with significantly reduced documentation of “delirium” in the discharge summary. This finding is unexpected but highlights the inaccuracies in discharge summary documentation for patients with delirium. Delirium is a syndrome that occurs secondary to another physiological, environmental or drug-induced insult, and therefore, after the work-up of delirium is completed, reasonably, the primary issue may receive greater emphasis in the discharge summary. Despite this, we believe delirium and specifically using the term “delirium” should still be documented in the discharge summary.

### Limitations

Limitations of our retrospective study include its inability to identify all cases of delirium within a time frame. Although the CHART-DEL is validated for identifying delirium with a specificity of 83 % [25], it relies on accurate chart reporting throughout hospitalization. This omits patients with insufficient delirium documentation and lowers our overall reported rates of delirium and possibly increases our reported rates of delirium documentation in discharge summaries. We recognize as well that varying hospital practices, such as mandatory delirium screening with a validated tool or routine involvement of geriatricians, could influence the rates of delirium documentation through improved recognition. There could have been an association between accurate delirium documentation in the chart and discharge documentation frequency or quality, but this relationship could not be elucidated as our methodology does not capture patients with delirium but poor chart documentation.

We acknowledge our methodology represents only a selected patient sample presenting with more severe or hyperactive delirium, and therefore greater chart documentation of confusion symptoms than compared to patients with less severe or hypoactive delirium. However, 30.9% of our patients were identified to have hypoactive delirium and 25.0 % had hyperactive delirium. This proportion is similar to previous proportions reported in previous studies [42–44]. We did not include an assessment of delirium severity in our data collection.

 Due to the time required for chart review, our sample size was modest, which limited our subgroup analysis (i.e. medicine vs. surgery) and prohibited a multivariable analysis that would produce meaningful findings. In assessing factors impacting documentation, our target sample size was not powered for our univariable regression analysis; rather only to analyze our primary outcomes. Additionally, these results should be interpreted in the setting of our selected patient population, whose delirium was detected and therefore adequately documented in the chart. Consequently, the results of our univariable regression model investigating variables associated with “delirium” documentation in the discharge summary (Table 4) must be viewed as hypothesis-generating and interpreted carefully.

Lastly, we recognize in the United States, documenting “metabolic encephalopathy”, rather than “delirium”, is preferential due to billing and reimbursement purposes [29]. In Canada, delirium does not affect funding in this manner. Though our primary outcome assessed for the term “delirium”, the CHART-DEL also accepts “metabolic encephalopathy” as an alternate term. Though we did not observe use of this term, our methodology would have captured charts documenting ‘metabolic encephalopathy’ within the subset of discharge summaries with “delirium” or another term accepted.

## Conclusions

Our study of patients with documented evidence of delirium in the medical record demonstrates higher rates of delirium documentation in the discharge summary compared to previous. However, multiple gaps remain, and improvements are necessary to meet quality standards. Although medical services document delirium with greater quality than surgical services, both services frequently lack detail needed to ensure comprehensive follow-up. Strategies to improve the frequency and quality of delirium documentation include use of templates and formalized education plans. Further work is required to determine the impact of delirium documentation on patient outcomes.

## Supplementary information

**Additional file 1.** Application of the Chart-Based Delirium Identification Instrument (CHART-DEL). This file goes into detail on how the CHART-DEL was applied to patient charts and outlines the selection of patients based on their probability of delirium based on this screening method. It also describes other information collected, as suggested by the CHART-DEL training manual, which we have referenced.

**Additional file 2.** Definitions of quality components used to assess quality of delirium documentation in the discharge summary. This file is a supplement to Table 3, as it provides definitions for each quality component. It explains the rationale for the differences in denominators when assessing various quality components.

## Data Availability

The datasets generated and analyzed during the current study are available from the corresponding author on reasonable request. We did not use any dataset from publicly available data.

## List of abbreviations

NICE: National Institute for Health and Care Excellence
PCP: Primary care provider
ICU: Intensive care unit
CHART-DEL: Chart-Based Delirium Identification Instrument
JCAHO: Joint Commission on Accreditation of Healthcare Organizations
HPI: History of presenting illness
