# Supplementary material for: The frequency and quality of delirium documentation in discharge summaries
Source: BMC Geriatr. 2021 May 12;21:307. doi: 10.1186/s12877-021-02245-3 (PMC8117503; doi:10.1186/s12877-021-02245-3)
Supplement: Supplementary file 1 — Additional file 1. Application of the Chart-Based Delirium Identification Instrument (CHART-DEL). This file goes into detail on how the CHART-DEL was applied to patient charts and outlines the selection of patients based on their probability of delirium based on this screening method. It also describes other information collected, as suggested by the CHART-DEL training manual, which we have referenced. [file 12877_2021_2245_MOESM1_ESM.docx]

**Additional File 1. Application of the Chart-Based Delirium Identification Instrument (CHART-DEL)**

We applied the CHART-DEL to all patients meeting initial screening criteria (n=1 1168). This involved reviewing all written materials from the medical record (e.g. emergency room note, admission note, progress notes, nursing and allied health notes, discharge summaries) for evidence of delirium and the patient’s baseline cognitive status. Trigger words and phrases to identify delirium as outlined in the CHART-DEL manual(26) were used to identify potential delirium. Once each chart review was completed, it was graded as “Definite”, “Probable”, “Possible” or “Uncertain” delirium. We included “Definite” and “Probable” cases only, which had a level of probability for delirium of ≥85% and 60-85% respectively.

“Definite” cases included patients who received a confirmed diagnosis by an experienced rater during hospitalization. “Probable” cases included patients who had all confusion assessment method (CAM) features present in the chart, and evidence of reversibility.(26) “Possible” cases included patients who had met at least two CAM features, with other supporting behaviours and “Uncertain” cases included patients who only had vague documentation pertaining to confusion in the chart.

Other data such as the onset of delirium, total days of delirium, type of delirium (e.g. hyperactive, hypoactive or mixed) and reversibility or improvement of delirium were also collected during this initial screening process and later used to identify appropriate documentation in the discharge summary.
