# Supplementary material for: The frequency and quality of delirium documentation in discharge summaries
Source: BMC Geriatr. 2021 May 12;21:307. doi: 10.1186/s12877-021-02245-3 (PMC8117503; doi:10.1186/s12877-021-02245-3)
Supplement: Supplementary file 2 — Additional file 2. Definitions of quality components used to assess quality of delirium documentation in the discharge summary. This file is a supplement to Table 3, as it provides definitions for each quality component. It explains the rationale for the differences in denominators when assessing various quality components. [file 12877_2021_2245_MOESM2_ESM.docx]

**Additional File 2.** Definitions of quality components used to assess quality of delirium documentation in the discharge summary

| **Quality Component** | **Study Definition** |
| --- | --- |
| Documented as chief complaint or history of presenting illness | Defines whether delirium was documented as part of the chief complaint or in the history of presenting illness in the discharge summary.  Applied only to patients who presented to hospital with delirium or its symptoms as a chief complaint or within their history of presenting illness. |
| Documented as diagnosis | Defines whether delirium was documented as a diagnosis anywhere in the discharge summary. Differentiates it by “delirium”, other terms or no documentation in the discharge summary.  Acceptable means of identifying delirium specifically as a diagnosis included: documentation as a listed primary or secondary diagnosis, documented in problem list or paragraph focused on delirium. It was not considered a documented diagnosis if delirium was mentioned in a larger paragraph.  Applied to all patients in the study. |
| Documented as 1º diagnosis | Defines whether delirium was appropriately documented as the primary diagnosis in the discharge summary.  Applied only to patients where delirium was the reason for admission to hospital. |
| Documented as 2º diagnosis | Defines whether delirium was appropriately documented as a secondary diagnosis in the discharge summary.  Applied only to patients where delirium was not the primary reason for admission but was present during hospitalization. |
| Documented in problem list | Defines whether delirium was documented in a problem list format in the discharge summary.  Applied to all patients in the study |
| Documented delirium onset | Defines whether the onset of delirium was documented in the discharge summary.  Applied to all patients in the study. |
| Documented delirium etiology | Defines whether the underlying etiology of delirium identified by the care team was documented in the discharge summary.  Applied only to patients whose etiology of delirium was identified and documented in the medical chart by the care team. |
| Documented consulting service involvement | Defines whether involvement of a consulting service for the delirium was documented in the discharge summary.  Applied only to patients who received specialist consultation for delirium. |
| Documented delirium work-up | Defines whether the work-up completed for the delirium was documented in the discharge summary.  Applied only to patients with delirium where a work-up to identify the underlying etiology was completed. |
| Documented delirium treatment | Defines whether the treatment for the underlying etiology of delirium was documented in the discharge summary.  Applied only to patients whose underlying etiology for delirium was identified and therefore, treatable. |
| Documented delirium status at discharge | Defines whether the status of delirium was documented in the discharge summary (i.e. resolved, unresolved)  Applied only to patients where the resolution of delirium could be identified in the medical chart. |
| Documented functional status at discharge | Defines whether the functional status (i.e. activities of daily living, mobility) at the time of discharge was documented in the discharge summary.  Applied to all patients in the study. |
| Documented relevant medication changes | Defines whether the medication changes related to delirium were documented in the discharge summary.  Applied only to patients who had medication changes related to their delirium management. |
| Documented reasons for medication changes | Defines whether the reason for medication change related to delirium was documented in the discharge summary.  Applied only to patients who had medication changes related to their delirium management. |
| Documented patient or family instructions | Defines whether patient or family instructions related to delirium were documented in the discharge summary.  Applied to all patients in the study. |
| Documented any follow-up recommendations | Defines whether the recommended follow-up of delirium was documented in the discharge summary.  Applied to any patient who had any ≥1 of cognitive, medication or specialist follow-up recommended, as evidenced through documentation in their medical chart. |
| Documented follow-up for psychoactive medications | Defines whether the recommended follow-up on psychoactive medications for delirium was documented in the discharge summary.  Applied only to patients who had received changes to their medications and follow-up was recommended by a physician as evidenced through documentation in their medical chart. |
| Documented follow-up for cognitive status | Defines whether the recommended follow-up on the patient’s cognition was documented in the discharge summary.  Applied only to patients who had received recommendations to have their cognition followed up by a physician, as evidenced through documentation in their medical chart. |
| Documented follow-up with specialist | Defines whether the recommended delirium follow-up with a specialist was documented in the discharge summary.  Applied to any patient who had received recommendations to have their delirium followed up by a specialist, as evidenced through documentation in their medical chart. |
| Copied to primary care provider | Defines whether the discharge summary was copied to the primary care provider (ie. family physician).  Applied to all patients in the study. |
| Signed by author | Defines whether the discharge summary was signed by the author of the discharge summary.  Applied to all patients in the study. |
